# Determination of Deoxynivalenol in the Urine of Pregnant Women in the UK

**DOI:** 10.3390/toxins8110306

**Published:** 2016-10-25

**Authors:** Liz Wells, Laura Hardie, Courtney Williams, Kay White, Yunru Liu, Barbara De Santis, Francesca Debegnach, Georgio Moretti, Stephanie Greetham, Carlo Brera, Alan Rigby, Stephen Atkin, Thozhukat Sathyapalan

**Affiliations:** 1 Department of Diabetes, Endocrinology and Metabolism, Brocklehurst Building, Hull Royal Infirmary, Anlaby Road, Hull HU3 2RW, UK; Thozhukat.Sathyapalan@hyms.ac.uk; 2 Division of Epidemiology and Biostatistics, LICAMM, School of Medicine, University of Leeds, Leeds LS2 9JT, UK; L.J.Hardie@leeds.ac.uk (L.H.); medcwila@leeds.ac.uk (C.W.); K.L.M.White@leeds.ac.uk (K.W.); 3 Department of Environmental Medicine, Hainan Medical University, 3 Xueyuan Road, Haikou 571199, China; liuyunru@126.com; 4 Department of Veterinary Public Health and Food Safety, GMO and Mycotoxins Unit, Istituto Superiore di Sanità, Viale Regina Elena, 299,00161 Rome, Italy; barbara.desantis@iss.it (B.D.S.); francesca.debegnach@iss.it (F.D.); carlo.brera@iss.it (C.B.); 5 Department of Statistical Sciences, Piazzale Aldo Moro, Università degli studi di Roma “La Sapienza”, 5,00185 Roma, Italy; giorgio.moretti87@gmail.com; 6 Echuca Regional Health, Service Street, Echuca 3564, Australia; sgreetham@erh.org.au; 7 Centre for Cardiovascular and Metabolic Research, Hull York Medical School, Hertford Building, University of Hull, Hull HU6 7RX, UK; asr1960@hotmail.com; 8 Weill Cornell Medicine in Qatar, Education City, P.O. Box 24144, Qatar; sla2002@qatar-med.cornell.edu

**Keywords:** mycotoxins, Deoxynivalenol, pregnancy, vomitoxin

## Abstract

Deoxynivalenol (DON) is one of the most commonly occurring trichothecenes, produced mainly by *Fusarium graminearum*. Little is known about the effect of DON exposure or the levels of DON exposure that occur during pregnancy. The project aimed to provide data on levels of total DON and de-epoxi Deoxynivalenol (DOM-1) in pregnant human urine samples analysed by liquid chromatography-mass spectrometry (LC-MS). Morning urine samples were collected over two consecutive days from 42 volunteers and associated food consumption was recorded for the 24 h prior to the sample. Spearman’s rho non-parametric test for correlation was used to assess the data. Levels of DON did not differ significantly between day 1 (mean 29.7 ng/mL urine or 40.1 ng DON/mg creatinine) and day 2 (mean 28.7 ng/mL urine or 38.8 ng DON/mg creatinine ng/mL/day) urine samples. The only significant positive correlation was found between total ng DON/mg creatinine and parity (rho = 0.307, *n* = 42, *p <* 0.005 two-tailed) and total ng DON/mg creatinine with baked goods on day 1 (rho = 0.532, *n* = 42, *p <* 0.0005 two-tailed). This study provides data on the DON levels in pregnancy in this suburban population and reassurance that those levels are within acceptable limits.

## 1. Introduction

Moulds of the genera *Aspergillus*, *Penicillium* and *Fusarium* naturally occur in grains and produce mycotoxins that can contaminate cereal-based food and foodstuffs. Deoxynivalenol (DON) belongs to a large group of mycotoxins named trichothecenes, which represent the main group of *Fusarium* toxins commonly found in cereal grains. Exposure assessment is an essential part of a risk assessment of xenobiotic compounds (e.g., mycotoxins) through diets containing contaminated food. Cereal-based foodstuffs are considered a major contributor to DON exposure. Therefore, the evaluation of DON and DON metabolites in urine may constitute a valuable indicator of the dietary exposure. On the basis of the available information derived from the toxicity studies, a temporary tolerable daily intake (TDI) of 1 μg/kg body weight (b.w.) was established by the EU Scientific Committee on Food [1] which was in line with the temporary tolerable daily intake established by the Nordic Group [2] and World Health Organization [3]. In 2010, the Joint Expert Committee for Food Additives (JECFA) [4] stated that, since metabolic studies indicated that DON acetylated forms are rapidly and extensively de-acetylated to DON, contributing to the total DON-induced toxicity, the provisional maximum tolerable daily intake (PMTDI) for DON could be converted to a group PMTDI of 1 μg/kg b.w. for DON and its acetylated derivatives, 3-acetyl-deoxynivalenol (3Ac-DON) and 15-acetyl-deoxynivalenol (15Ac-DON) [4]. The Committee also concluded that there was insufficient information to include DON-3-glucoside in the group PMTDI. JECFA concluded also that DON is a probable factor for acute pathologies in humans and derived an Acute Reference Dose (ARfD) of 8 μg/kg b.w.

Recent studies have demonstrated that consumption of DON-contaminated grain-based products is associated with the presence of DON and its metabolites (e.g., DON-glucuronide) in human urine. The available data show that the main fraction of this mycotoxin is excreted in the urine. DON is detectable also in serum in high amounts immediately after ingestion, but is rapidly cleared from the blood stream. So far, three major metabolites of DON have been identified in mammals: namely, deoxynivalenol-3-glucuronide (DON-3-GlcA), deoxynivalenol-15-glucuronide (DON-15-GlcA) and de-epoxi Deoxynivalenol, also known as DOM-1. Analytical methods with high sensitivity have been developed and applied for precise quantification of DON and/or its metabolites in urine samples [5].

Little is known about the effect of DON exposure during pregnancy on the human foetus or the levels of DON exposure that occur during pregnancy. During pregnancy the foetus is exposed to the contaminants contained in the mothers’ diet. DON is known to cause a range of adverse effects in animals and can cross the placental barrier. Research has shown that DON can transfer to the foetus of pregnant sows [6] and has been linked to restrictions in both growth and immune function [7]. In swine, DON intake reduces weight gain and hinders animal feeding. Acute exposure of pigs to DON at high concentrations (more than 10 mg/kg) causes abdominal distress, increased salivation, malaise, diarrhoea, emesis [8,9] and some feed refusal [10]. Due to these effects, DON is also known as vomitoxin. Given that DON can cross the placenta of animals [6] it is likely that in utero exposure to DON will occur in humans. The detoxification capacity of the foetus will not be fully developed, at a time of rapid growth and cell turnover [11], therefore pregnancy may represent a critical window for DON exposure. Thus, the exposure of pregnant women to DON in the diet is of concern. This paper affords a distinctive data set detailing the DON exposure of White Caucasian pregnant women in a suburban setting, including females in their first trimester of pregnancy, providing data on levels of DON in human urine samples collected from pregnant women in Hull, East Yorkshire, as analysed by liquid chromatography-mass spectrometry (LC-MS).

## 2. Results

A total of 42 White Caucasian females were enrolled in the trial between May and October of 2014. Ages ranged from 20 to 38 years, with a mean of 28 years, and a standard deviation (SD) ±4.6. The majority of females were in the second trimester of pregnancy (*n* = 23) with a mean gestational age of 26 weeks. Only two females were in the first trimester and 17 were in the final trimester. Of the 42 pregnant women, 19 (45%) were pregnant for the first time, 13 (31%) had one previous pregnancy, six (14%) had two previous pregnancies, three (7%) had three previous pregnancies and one (2%) had experienced four or more previous pregnancies. The mean height was 1.65 m, with a range of 1.49–1.77 m, (SD) ±0.065, and the mean weight was 84 kg, with a range of 59–150 kg, (SD) ±19.47; this equated to a mean BMI of 32 kg/m^2^ with a large range of 21–57 kg/m^2^, (SD) ± 7.2. 

Free DON (>LOD 0.12 ng/mL), and DON glucuronide (>LOD 0.25 ng/mL) were detected in most of the urine specimens, whereas DOM-1 and de-epoxy DON glucuronide were not detected (LOD 0.25 ng/mL).

**Figure 1:**
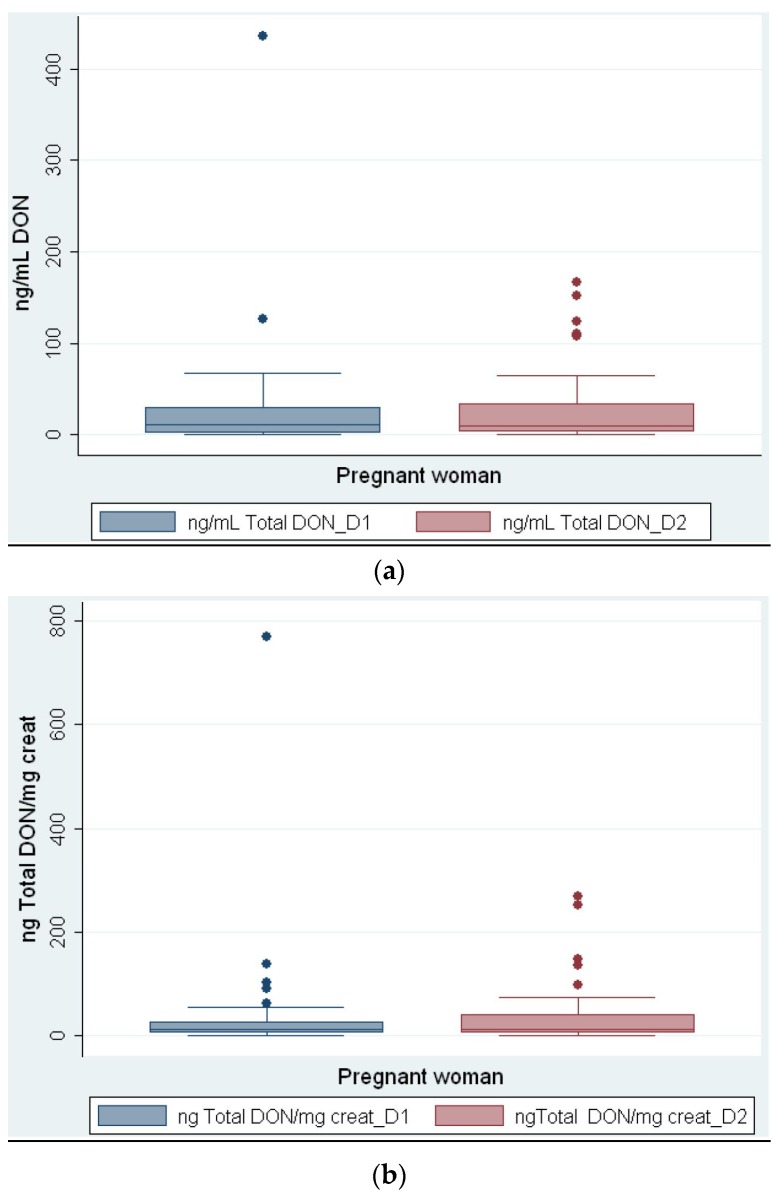
Box plot of (**a**) unadjusted and (**b**) creatinine-adjusted total DON concentrations in pregnant women’s urine samples for day 1 (blue) and day 2 (red) [12]. The band inside the box is the second quartile (P50, median). Dots are suspected outliers. Whiskers are set from minimum to maximum value. The bottom and the top of the box are the first and third quartiles (P25 and P75).

Total DON (free DON and DON glucuronide combined) was detected in 88.1% (*n* = 37) of women on day 1 and 83.3% (*n* = 35) on day 2. With respect to total DON, free DON and DON glucuronide represented 12% and 88%, respectively, on day 1, and 16% and 84%, respectively, on day 2. Only three of the pregnant females had no DON present in their urine on day 1 and day 2 of the study. Four of the pregnant females had DON in their urine on day 1 of the study but not on day 2 of the study and, conversely, two of the pregnant females had DON in their urine on day 2 of the study but not on day 1 of the study. The mean values were obtained using the lower bound approach. On day 1 the mean level of total DON (ng/mL urine) (*n* = 42) was 29.7 (range 0–436), and on day 2 it was 28.7 (range 0–167) (See Figure 1a). In order to adjust the urinary total DON levels (ng/mL) for the effects of fluid balance, the ng/mL of total DON was corrected for mg/mL of creatinine. Therefore, on day 1 the mean level of ng total DON/mg creatinine (*n* = 42) was 40.1 (range 0–769), and on day 2 it was 38.8 (range 0–269) (See Figure 1b).

Due to the skewed nature of the data, non-parametric tests for correlation (Spearman’s rho) were used for analysis. Tests for total DON (ng/mg creatinine) vs. weight, age, BMI, physical activity, week of gestation, trimester and parity were performed. A significant positive correlation was only found between total DON (ng/mg creatinine) and parity (rho = 0.307, *n* = 42, *p <* 0.005 two-tailed). Generic associations between food consumption and urinary DON were assessed by ordered logistic regression models. Tests were performed for total DON (ng/mg creatinine) vs. food intake (grams) for each of the seven food categories on day 1 and day 2. The only significant correlation was found between total DON (ng/mg creatinine) on day 1 with baked goods (rho = 0.532, *n* = 42, *p <* 0.0005 two-tailed). Baked goods were defined as sweet biscuits such as digestives or hobnobs excluding fine bakery wares such as croissants and cakes. Only one participant, a female, followed a vegetarian diet.

Based on the daily food diaries documented by participants in the 24 h prior to the first morning’s urine sample, the most common cereal choice for pregnant females in Hull was wheat bran flakes. Porridge high in bran was never consumed. Of the seven categories, bread provided the largest contributor to the daily food intake, followed by pasta, then baked goods. Of the category “products alternative to bread”, pizza was the most commonly consumed food. Plain dried (durum wheat) pasta was the most commonly consumed food in the pasta category. Fresh egg pasta was the least consumed food in the pasta category. No beer was consumed by any of the participants on day 1. On day 2, two participants consumed lager; one consumed a medium portion (574 g = 1 pint) and one consumed a small portion (287 g = 12 pint) [13]. On analysis of the Food frequency questionnaires (FFQ), alcohol-free lager was more commonly consumed in comparison to standard lager.

## 3. Discussion

Mycotoxins contaminate up to 25% of the world’s cereal crops; the potential health consequences of these exposures remain mostly poorly investigated [14]. The high incidence of DON in the urine of UK pregnant females in this study (88% on day one and 83% on day two) confirms its ubiquitous presence in cereal food products in the UK. This study provides distinctive findings describing the level of DON in the urine of a cohort of White Caucasian pregnant women residing in Hull, East Yorkshire, UK, at mean levels of 40.1 ng and 38.8 ng/mg creatinine on two separate days that are within the recommended TDI [1]. 

Human research involving pregnant women in Bradford, West Yorkshire (60 miles west of the current study), was carried out on 85 pregnant women as part of the Born in Bradford study (BIB). The females provided urine samples during the last trimester of pregnancy; 34% of the women were classified as being of South Asian origin. The distribution of urinary DON was similar to that reported here, although some of the readings were the highest ever recorded in a study [15]. The GM urinary DON level was 10.3 ng/mg creatinine (range of 0.5–43.3), with a marked difference between women of South Asian origin where the GM was 15.2 compared to the non–South Asian GM of 8.6 ng DON/mg creatinine, respectively [15]. In the Turner et al.’s (2008) study of the UK adult population, the GM level of urinary DON after adjustment for creatinine was 8.9 ng/mg (95% CI, 8.2–9.7 ng DON/mg creatinine; range 0.6–48.2 [16]. The GM for the Hull females was 11.72 ng DON/mg creatinine (range of 0.01–769), similar to that in Bradford of 10.3 ng/mg creatinine [15]. It is suggested that a mean urinary level of 9–10 ng DON/mg creatinine corresponded to an intake of roughly 20%–25% of the recommended tolerable daily intake (rTDI) of 1000 ng/kg b.w. [1]. From this we can extrapolate that, on average, the pregnant females in Hull were likely to be consuming safe levels of DON in their diet. 

DON exposure may differ around the world. In Bangladesh, researchers found DON exposure in pregnant women was significantly lower and more modest than previous results from Europe or Asia [17]. This in part may be due to their diet habits, but it could not be deduced as FFQs were designed to capture aflatoxins rather than DON, stating only that the contributors would predominantly include wheat and maize if surveyed [18]. Piekkola et al. (2012) performed a cross-sectional assessment of 98 women, in their third trimester of pregnancy in rural Egypt [18], providing descriptive data on the concentrations of DON, DOM-1 and aflatoxin biomarkers in urine and blood. This was the first work of its kind to be carried out on an African population. They found the frequency and levels of these biomarkers in Egyptian women were modest, compared to other high-risk areas. DON was only observed in 68% of the pregnant women’s urine and the GM of DON was 1.11 ng/mg creatinine (range 0.6–24.3 ng/mg creatinine). These findings represent the lowest GM DON/creatinine of all the findings discussed in this paper. The detection of DON in urine was also low in comparison to other studies and similar to that of levels found in rural Bangladesh [16]. 

Pregnant females of Asian descent, living in suburban areas, appear to the highest levels of GM DON/creatinine in their urine levels [15]. It is unclear whether this is due in some part to ethnicity, living in a suburban area, diet consumed or all of the above. Pregnant women from the suburban areas of Bangladesh had a significantly higher mean level of urinary DON (1.44 ± 2.20 ng/mL) than their rural counterparts (0.47 ± 0.73 ng/mL). The researchers [16] attribute this to a higher consumption of wheat breads in the suburban participants. It is suggested that white, wholemeal and other bread cereals contributed significantly to DON levels in the diets of pregnant and non-pregnant females from the general UK population [5,15]. The higher intake of chapattis by women of South Asian origin was attributed to the higher level of DON when compared with women of non-South Asian origin (GM 15.2 vs. 8.6 ng DON/mg creatinine, respectively). For the pregnant females in Hull the only significant correlation was found between ng total DON/mg creatinine on day 1 with baked goods [12]. Baked goods are defined as sweet biscuits such as digestives or hobnobs, excluding fine bakery wares such as croissants and cakes.

Adjusting for individual bodyweight and assuming daily urine excretion of 2 L and DON excretion rates of 68%, the provisional daily intake (PDI ng/kg b.w.) could be assessed utilizing the calculations described by Ali et al. [16]. Urinary DON levels were much higher in pregnant women from Hull compared to Bangladeshi ones (both from rural and suburban areas): values of 29.7 ng/mL, (SD) ±69.3, for day 1 and 28.7 ng/mL, (SD) ±42.5, for day 2 in Hull when compared with average values of 0.86 ng/mL, (SD) ±1.57, in Bangladesh. Consequently, mean PDI levels were much higher for Hull than for Bangladeshi women. Therefore, while the entire population in Bangladesh would be far from the rTDI of 1000 ng/kg b.w., 25% of the Hull group would exceed the rTDI according to these calculations (75th percentile being 1012.4 ng/kg b.w. for day 1 and 1254.8 ng/kg b.w. for day 2) and the 95th percentile would exceed the rTDI by a factor of three times and five times for day 1 and day 2, respectively. This scenario may be a reason for concern, especially for this specific vulnerable group of the population, and may give the opportunity for reconsidering the toxicological threshold and/or legal limits. However, the 50th percentile of the Hull group (being set to 411.2 ng/kg b.w. for day 1 and 399.5 ng/kg b.w. for day 2) is below the rTDI. 

A 24 h food diary is a quick, cheap and simple method for performing dietary analysis. However, variability can occur between oral intakes day to day [19]. To counteract this, two consecutive 24 h food diaries were used for this study. A semi-quantitative FFQ was utilised to record the participants’ previous month’s food intake, portion size and whether or not the food was organic. Food frequency questionnaires are thought to provide estimates of habitual intake and are widely used in nutritional epidemiology [20,21]. The length and complexity of the FFQ can influence the response to dietary recording. Two advanced research dietitians were responsible for performing the semi-structured interviews on all participants in the study. The interviewing and counselling skills of the experienced research dietitians will have removed a degree of potential error from the data recorded. 

The limitations of the study include the small sample size of 42 women and no organic foods being eaten by the pregnant females in the study; consequently, organic food as a variable could not be analysed fully due to the absence of data. The lack of organic food consumed is likely to be secondary to the deprived nature of the population studied. In order to assess the effect of organic food consumption, a fit for purpose study should be designed to consider organic food consumers as a category. Portion size can represent a major source of uncertainty for food items contributing to DON exposure. Documentation of actual weights consumed could have improved the accuracy of the food data recorded ready for correlation with DON concentration in urine. To obtain a more accurate estimation of the carry over of DON from ingested food to urine, 24 h urine collection rather than a single first morning urine sample would be desirable.

Very few studies have been conducted so far in the population subgroup of pregnant women and little is known about the consequences of exposure. Future large-scale studies with multi-site analysis are required to give a more varied population sample to extrapolate results.

## 4. Conclusions

In conclusion, this study provides data on the DON levels in pregnancy in this suburban population and it is reassuring that 50th percentile is within acceptable and safe limits. However, it should be appropriate to consider a revision of the TDI and/or DON maximum limits and to set additional agricultural strategies to minimize the exposure, improving the quality of wheat raw materials. Finally, an appropriate information program given by physicians would help to orient the dietary habits of UK pregnant women and protect this vulnerable group, also in consideration of the potential of toxin transfer to the foetus.

## 5. Experimental Section

### 5.1. Ethics

Ethical approval was granted by the National Health Service (NHS), National Research Ethics Service (NRES) Committee Yorkshire & the Humber—Leeds West on the 11 April 2014. IRAS project code 147707. SPSS IBM 23 and Microsoft excel 2013 were used to analyse the data.

#### Recruitment

Pregnant women were recruited by accessing weekly antenatal clinics at Hull Royal Infirmary. Recruitment took place between May and October 2014. Participants were also recruited by placing an approved advertisement in the local papers and an email “shot” via the University of Hull and Hull and East Yorkshire Hospitals NHS portal.

The pregnant females were required to be in good health, taking no medication or on stable medication. For the purpose of this study, stable medication was defined as medication taken for more than three months, however the medication prescribed even if stable must not affect appetite, e.g., oral steroids. Exclusion criteria included inability to give informed consent/complete the questionnaire, acute pathologies, chronic illness (chronic renal, hepatic or cardiac problems, cancer), chronic gastrointestinal conditions (e.g., coeliac disease), gluten sensitivity, eating disorders, following a weight loss diet, depression, psychosis or hospitalisation within three months of enrolment in the trial.

### 5.2. Methods

The data utilised in this study is extracted from a larger study to answer a call from the European Food Safety Authority (EFSA) GP/EFSA/CONTAM/2013/04 into the *Experimental study of deoxynivalenol biomarkers in urine* [12]. The study collected data on the occurrence of DON and its metabolites in urine from European population groups, namely children, adolescents, adults, elderly, vegetarians (defined as following the diet for >1 year) and pregnant women and to develop reliable information on the associations between concentrations of DON in urine and self-reported cereal food consumption in the investigated subjects. A total of 635 volunteers were recruited across three European countries, i.e., Italy, Norway and the UK. This paper focuses on the pregnant subset of data in the UK.

Age, height, ethnicity, weight, physical activity level, week of gestation, parity, and any dietary restriction was recorded using FFQ. The FFQ comprised of 61 cereal-based food items, split into seven different food categories, all considered to contribute to the consumption of DON. The food categories included breakfast cereals and snacks, bread, products alternative to bread, pasta, biscuits and bakery products, cereals and similar and beer. The research dietitians conducted a semi structured interview to record the intake of each food category consumed over the last month, the size of said portion and whether the food was organic. The FFQ featured photographic examples of portion sizes to aid the participants in the quantification of the food consumed. The basis for the FFQ was the recent validated questionnaire used in a Spanish study targeted to pregnant women [22] and the National Health and Nutrition Examination Survey (NHANES) FFQ [23]. The urine collection method was explained to each participant by the Research dietitian or Research nurse. Participants were provided with a collection container, 4× urine sample pots (50 mL) and written instructions for urinary collection at home. The participants were asked to provide a sample of first morning urine, in their own home, on two consecutive days and decant the urine from the bottle into 2 × 50 mL pots on each day. A 24 h food diary was documented by the participant over two consecutive days (on the days prior to each collection of their first morning urine sample). The 24 h food diary consisted of sections for breakfast lunch, dinner and snacks. Within each meal group, examples of food taken from each food category were tabulated with tick boxes provided to allow ease of recording as to whether the portion was small, medium, large or organic. The urine samples were returned to the clinical trials unit on the same day and centrifuged immediately at the Hull Royal Infirmary (Hull, UK), in a refrigerated centrifuge at 2000 rpm for 10 min. The samples were then pipetted into a transfer container leaving a small amount of urine behind therefore excluding any debris. The samples were then frozen at −80 °C and the freezer temperature was monitored regularly. One sample from each day was sent to Leeds University (Leeds, UK) for analysis in batches and the other sample were stored at the clinical research unit in Hull as a reserve in case of loss or damage to the primary sample whilst being transported to Leeds University. 

Analysis of Urine samples for DON and its metabolites were carried out utilising the validated HPLC-MS/MS methodology developed at Leeds University [5]. The levels of the subjects in the UK were measured in urine samples in two steps. In brief, urine samples (mainly around 10 mL) were removed from storage at −20 °C, allowed to thaw, and centrifuged (2000 rpm; 15 min; 4 °C). Aliquots (1 mL) were mixed with ^13^C-DON internal standard solution, to give a final concentration of 20 ng/mL. 

#### 5.2.1. Aliquot 1

Total DON detection (free DON and glucuronide metabolites combined): To measure free DON and combined glucuronide metabolites of DON (deoxynivalenol-3-*O*-glucuronide and deoxynivalenol-15-*O*-glucuronide), each sample was adjusted to pH 6.8 and digested using β-glucuronidase solution (23,000 units, in KH_2_PO_4_ 75 mM) in a shaking water bath for 18 h at 37 °C. After this period, the samples were removed, centrifuged (2000 rpm; 15 min; 4 °C), and the supernatant diluted to a final 4 mL with phosphate buffered saline (PBS, pH 7.4). The diluted urine sample was then passed through a wide bore DON immunoaffinity column, following the manufacturer’ instructions. DON was eluted from columns with methanol (4 mL) and extracts dried under vacuum using a SavantTM SpeedVacTM (Thermo Fisher Scientific Inc., Waltham, MA, USA) or equivalent and reconstituted in 10% ethanol (250 μL) for LC-MS analysis. 

DOM-1 was quantified on the same aliquot analysed for DON glucuronide. In fact, the available data, coming from previous experience, indicate that urinary digestion using β-glucuronidase is able to release DOM-1 quantitatively.

#### 5.2.2. Aliquot 2

Free DON detection: the free amount of DON was extracted as before but omitting the β-glucuronidase treatment. 

#### 5.2.3. LC-MS Analysis—DON Determination

Separation of DON was achieved using reversed phase chromatographic columns and a mobile phase sequence of 27 min 20% methanol, changing to a wash of 75% methanol after 10 min and reverting to 20% methanol after 16 min (flow rate 1 mL/min; injection volume 25 μL). One-fifth of the eluent was directed into the desolvation chamber of the MS and the remainder pumped to waste. Selective ion recording (SIR) was used to quantify DON by reference to ^13^C-DON internal standard. Two masses both of DON ([DON-H]^+^, *m*/*z* 297.2 and [DON-Na]^+^, *m*/*z* 319.2) and ^13^C-DON ([^13^C-DON-H]^+^, *m*/*z* 312.2 and [^13^C-DON-Na]^+^, *m*/*z* 334.2) were monitored for 0.25 s each and summed to produce one total ion current peak for each analyte and internal standard. The calibration curve was set by the injection of DON and ^13^C-DON standard solution (prepared in 10% ethanol) covering the range 2–250 ng/mL. 

In addition to measuring free DON and total DON, DON glucuronide values were calculated as the difference between total DON and free DON values for each individual.

#### 5.2.4. LC-MS Analysis—DOM-1 Determination

Separation of DOM-1 was achieved using the same chromatographic column used for DON and a mobile phase sequence of 35 min 20% methanol, changing to a wash of 75% methanol after 20 min and reverting to 20% methanol after 26 min (flow rate 1 mL/min; injection volume 25 μL). One-fifth of the eluent was directed into the desolvation chamber of the MS and the remainder pumped to waste. SIR was used to quantify DOM-1 by reference to the calibration curve, obtained by the injection of DOM-1 standard solutions (prepared in 10% ethanol) covering the range 2–200 ng/mL, using least squares regression analysis. Two masses of DOM-1 ([DOM-1-H]^+^, *m*/*z* 281.3 and [DOM-1-Na]^+^, *m*/*z* 303.3) were monitored for 0.25 s each and summed to produce one total ion current peak for DOM-1.

#### 5.2.5. Creatinine Analysis

Since only first morning samples of urine were collected, levels of DON were adjusted for creatinine to correct for variable dilutions. Urinary creatinine analysis was conducted for all samples using an in-house micro-titre plate assay as previously described and modified from the alkaline-picrate method [15]. In brief, urine samples were diluted in water (1:20) and 100 µL loaded into a 96-well plate, in duplicate. A duplicate standard curve of creatinine concentrations was used per plate ranging from 0 µg/mL to 20 µg/mL. 100 µL of alkaline picric acid solution was then added to each well, incubated at 25 °C for 30 min and read at 490 nm using a plate spectrophotometer.

Results were presented both corrected and uncorrected for creatinine as Total DON ng/mL urine and as g total DON ng/mg creatinine in urine. The latter allow a correction for different degrees of dilution of the urine between individuals.

#### 5.2.6. Dietary Analysis

The research dietitians converted the portion sizes documented into the FFQ and food diaries to actual quantities (grams) prior to their entry into the FoodEx2 coding system [13,24]. FoodEx2 (revision 1) is a comprehensive food classification and description system aimed at covering the need to describe food in data collections across different food safety domains issued by EFSA. Central to the system is a core list of food items or generic food descriptions that represent the minimum level of detail needed for intake or exposure assessments [25]. The FoodEx2 code number for each food was retained and included in the database including the results from the FFQ. A harmonized database was developed to collect all the data related to the enrolled subjects concerning individual information (age, gender, BMI, and other information), urine testing results and food intake. In total, the UK FFQ comprised of 61 cereal based food items, each food corresponded to a FoodEx 2 code, e.g., A008K. 

## Abbreviations

The following abbreviations are used in this manuscript:

b.w.	Body weight
FFQ	Food frequency questionnaire
DON	Deoxynivalenol
EFSA	European Food Safety Authority
NHANES	National Health and Nutrition Examination Survey
BMI	Body Mass Index
DOM-1	De-epoxi Deoxynivalenol
rTDI	recommended tolerable daily intake
GM	Geometric mean
PMTDI	Provisional maximum tolerable daily intake
ARfD	Acute Reference Dose
LC-MS	liquid chromatography–mass spectrometry
UK	United Kingdom
